# Impact of face masks on voice radiationa)

**DOI:** 10.1121/10.0002853

**Published:** 2020-12-14

**Authors:** Christoph Pörschmann, Tim Lübeck, Johannes M. Arend

**Affiliations:** Institute of Communications Engineering, TH Köln—University of Applied Sciences, Betzdorfer Straße 2, 50679 Cologne, Germany

## Abstract

With the COVID-19 pandemic, the wearing of face masks covering mouth and nose has become ubiquitous all around the world. This study investigates the impact of typical face masks on voice radiation. To analyze the transmission loss caused by masks and the influence of masks on directivity, this study measured the full-spherical voice directivity of a dummy head with a mouth simulator covered with six masks of different types, i.e., medical masks, filtering facepiece respirator masks, and cloth face coverings. The results show a significant frequency-dependent transmission loss, which varies depending on the mask, especially above 2 kHz. Furthermore, the two facepiece respirator masks also significantly affect speech directivity, as determined by the directivity index (DI). Compared to the measurements without a mask, the DI deviates by up to 7 dB at frequencies above 3 kHz. For all other masks, the deviations are below 2 dB in all third-octave frequency bands.

## I. INTRODUCTION

In times of crisis, such as the COVID-19 pandemic, security measures affect human face-to-face communication in many different ways. Speech intelligibility is impaired by greater physical distances between interlocutors, resulting in increased masking by background noise and a lower direct-to-reverberant ratio (DRR) at the listener position. In addition, face masks covering the mouth and nose cause transmission loss, reducing the energy radiated by the speaker. Depending on the frequency characteristics of the transmission loss, this can affect speech intelligibility (Palmiero *et al.*, 2016; Radonovich *et al.*, 2010). As the transmission loss of the masks may also vary with respect to the direction of radiation, the DRR could be further affected if, for example, the masks attenuate frontal sound radiation more than lateral radiation. Furthermore, other effects (e.g., lip reading) also strongly affect speech intelligibility (Matthews *et al.*, 2002; Sumby and Pollack, 1954; Summerfield, 1992).

So far, the acoustic effects of face masks have only been investigated in a small number of studies. Radonovich *et al.* (2010) and Palmiero *et al.* (2016) evaluated the influence of respirator masks worn by healthcare workers on speech intelligibility. In these studies, the influence of masks on the speech transmission index (STI) was determined for certain room settings. Recently, Goldin *et al.* (2020) analyzed three medical masks and found a low-pass characteristic attenuating frequencies above 2 kHz.

To further investigate to what extent the acoustic effects of face masks affect voice radiation and consequently speech intelligibility, we present full-spherical voice directivity measurements of a dummy head with a mouth simulator covered with six masks of different types, i.e., medical masks, filtering facepiece respirator masks, and cloth face coverings. We analyze the transmission loss caused by the masks as well as their influence on directivity. Although it is already clear that the transmission loss caused by the masks can reduce speech intelligibility, it has not yet been investigated how face masks affect speech directivity and, therefore, possibly also speech intelligibility. Resonances of vibrating structures of the mask or cases where the mask is not completely closed at the sides may lead to reduced frontal sound radiation compared to the lateral or rear radiation, impairing speech directivity. Accordingly, when a speaker inside a room faces a listener, the reverberant energy caused by sound radiation in all directions is decreased less than the direct sound energy, resulting in a reduced DRR at the listener position and possibly reduced speech intelligibility. Furthermore, the influence on the directivity could become relevant if the interlocutors do not look at each other directly but turn their heads slightly and thus radiate speech laterally, as is often observed in conversations.

## II. MEASUREMENT PROCEDURE

The directivity measurements were done with a HEAD acoustics HMS II.3 dummy head and mouth simulator (head width 14.0 cm; head height 22.5 cm; head length 20.0 cm). However, as a mouth simulator cannot reflect phoneme-dependent effects, there are distinct differences between the radiation of a dummy head and the human voice. For example, the mouth opening's variable size plays an important role (Brandner *et al.*, 2020), as well as sound radiation of the nasal passage and the position of the voice excitation in the vocal tract. These phoneme-dependent effects are one important aspect of the dynamic voice directivity which has been analyzed in several studies, e.g., in Kocon and Monson (2018), Katz and D'Alessandro (2007), Monson *et al.* (2012), and Pörschmann and Arend (2020). While simulations could probably examine some aspects of voice radiation such as the mouth opening's variable size, other aspects such as the physical contact between the mask and the vibrating lips are much harder to analyze. Despite these differences, a dummy head's radiation generally covers typical spatial characteristics of human voice radiation (Halkosaari *et al.*, 2005) and therefore provides a good approximation. Accordingly, we assume that the influence of the masks on voice radiation is comparable for a dummy head and human voice radiation.

**FIG. 1.**
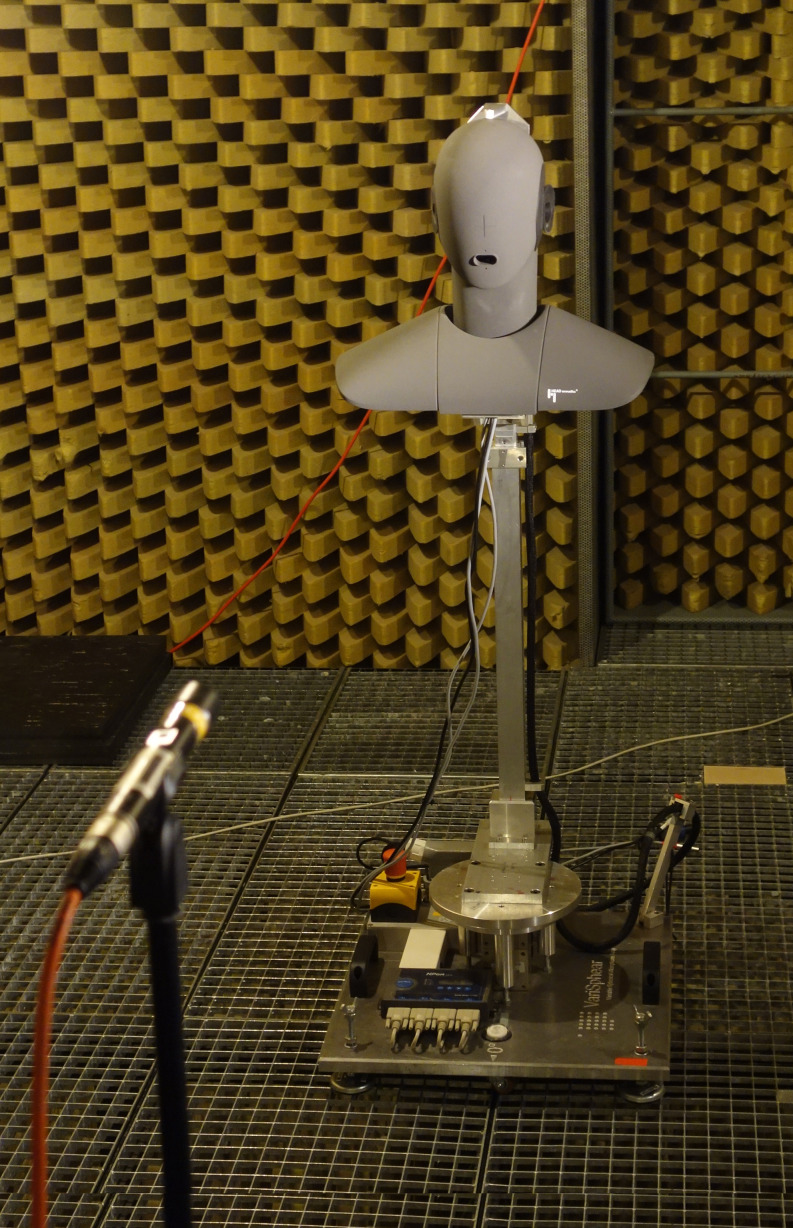
(Color online) Measurement setup with the HEAD acoustics HMS II.3 dummy head and mouth simulator mounted on the VariSphear measurement system (Bernschütz *et al.*, 2010) and the omnidirectional Microtech Gefell M296S measurement microphone at a distance of 2 m from the dummy head.

The measurements were performed in the anechoic chamber of TH Köln, sized 4.5 m × 11.7 m × 2.30 m (W × D×H), with a lower boundary frequency of about 200 Hz. As shown in Fig. 1, the HEAD acoustics HMS II.3 dummy head and mouth simulator was mounted on the VariSphear measurement system (Bernschütz *et al.*, 2010), which rotated the dummy head along a virtual sphere to the respective direction. In each direction, the excitation signal was played back over the mouth simulator and captured at a fixed position at a distance of 2 m from the center of the dummy head with a Microtech Gefell M296S omnidirectional microphone. The loudspeaker (mouth simulator) of the HEAD acoustics HMS II.3 was driven by an Apart MB-150 amplifier. An RME Babyface was used as AD/DA converter and microphone amplifier. The excitation signal was an emphasized sine sweep (2^18^ samples at a sampling rate of 48 kHz, length of 5.5 s). Impulse responses were measured for 2702 directions on a 44th order Lebedev sampling grid (Lebedev, 1977). In post-processing, adaptive low-frequency extension for frequencies below 200 Hz was applied to the impulse responses (Xie, 2009). Subsequently, the frequency and phase response of the loudspeaker (mouth simulator) was compensated by inverse finite impulse response (FIR) filtering with a frontal impulse response measured without a mask at azimuth ϕ=0° and elevation θ=0°, corresponding to a free-field equalization of the measurements. Finally, the impulse responses were truncated and windowed to a length of 128 samples at a sampling rate of 48 kHz.

Except for the reference directivity measurement of the mouth simulator without a mask, the measurement procedure was repeated twice for each of the following face masks:

(1) Disposable medical mask: Triple Layer Filter, dustproof, non woven earloop, (manufactured in China, brandname: Arvin, Protection Class DS3).
(2) Three-dimensional respirator mask, Protection Class KN 95 (manufacturer: Suzhou Jinruida Protective Equipment Co, Inc.).
(3) Fine dust respirator mask, MB 21, Protection Class FFP 2 (manufacturer: MB Filter Products AB, Sävedalen, Sweden).
(4) Microfibre scarf (manufacturer: Rose, material: 100% polyester).
(5) Cloth face covering: Single layer cotton (manufacturer: Modeatelier Scharn, Engelskirchen, Germany).
(6) Cloth face covering: Hand-made with two layers of cotton (manufacturer: Stoffliebe, Gelsenkirchen, Germany).

**FIG. 2.**
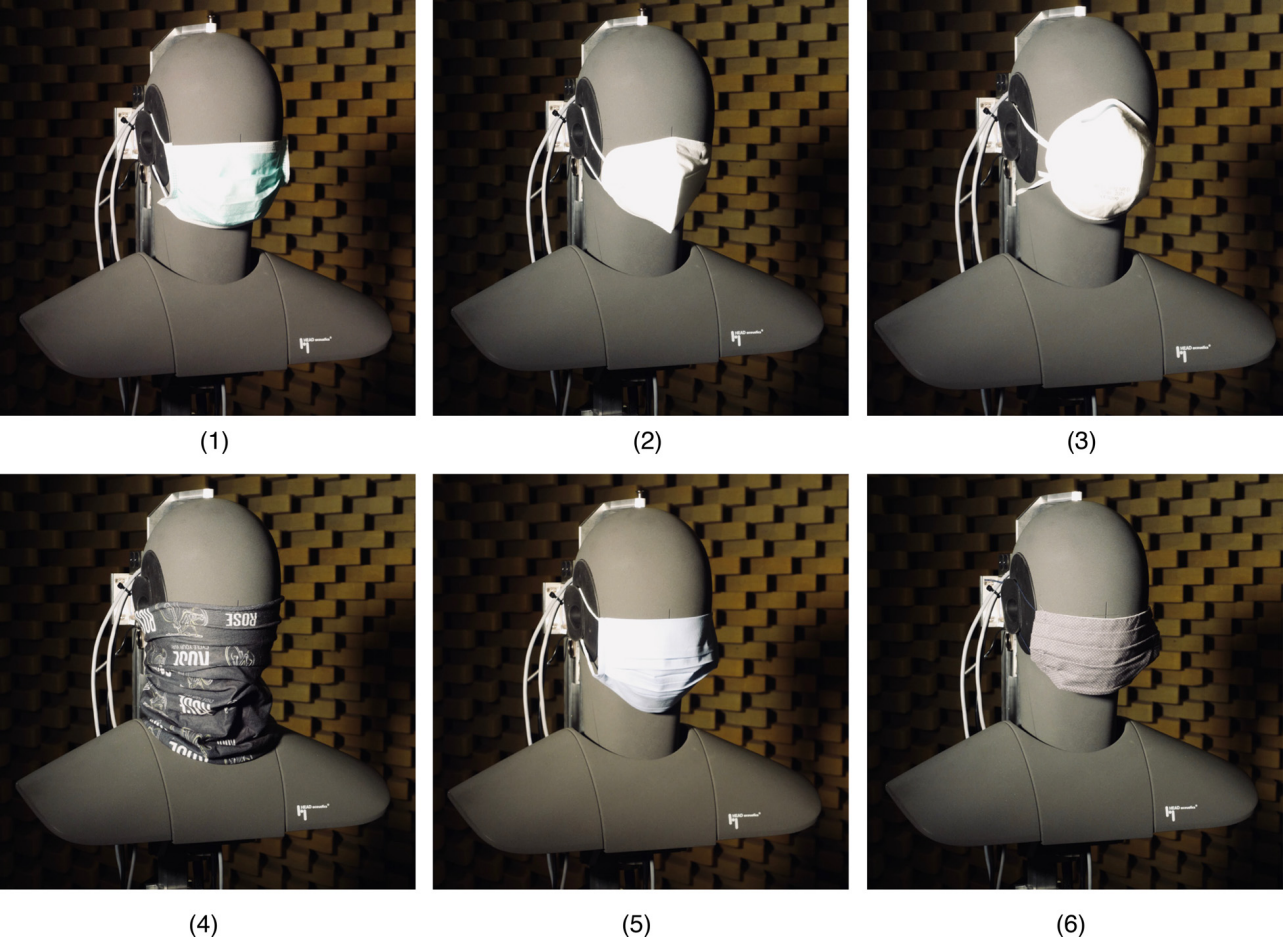
(Color online) HEAD acoustics HMS II.3 dummy head and mouth simulator with all the masks tested.

According to the World Health Organization (2020), these masks (or more generally mouth and nose covers) can be categorized as medical masks (1), filtering facepiece respirator masks (2, 3), or cloth face coverings (4, 5, 6). Figure 2 shows the dummy head with the six tested masks.

## III. RESULTS

Every mask was measured twice. In between, it was taken off and put on again. We calculated the signed difference between both measurements in dB averaged over all directions for frequencies below 8 kHz to analyze any possible deviations. The deviations were only about 0.5 to 0.8 dB, so we did not further investigate the effects of putting on and taking off the mask and simply refer to the first measurements in the following. However, the publicly available dataset contains all measurements.^1^

### A. Transmission loss

In the remainder, the directivity is denoted as p(Ω,ω) with respect to the direction Ω (ϕ, *θ*) and the angular frequency ω=2πf, where *f* is the temporal frequency. ϕ denotes the azimuth angle ranging from −180° to +180°, and *θ* the elevation ranging from −90° to 90°, where 90° is at the top, and −90° at the bottom. The transmission loss T(Ω,ω) between the reference directivity $p_{\text{ref}}(\Omega ,\omega )$ without a mask, and the directivity $p_{\text{mask}}(\Omega ,\omega )$ with a mask, can be expressed as

$$T(\Omega ,\omega )=10\text{lg}\frac{|p_{\text{ref}}(\Omega ,\omega ){|}^{2}}{|p_{\text{mask}}(\Omega ,\omega ){|}^{2}}.$$ (1)

**FIG. 3.**
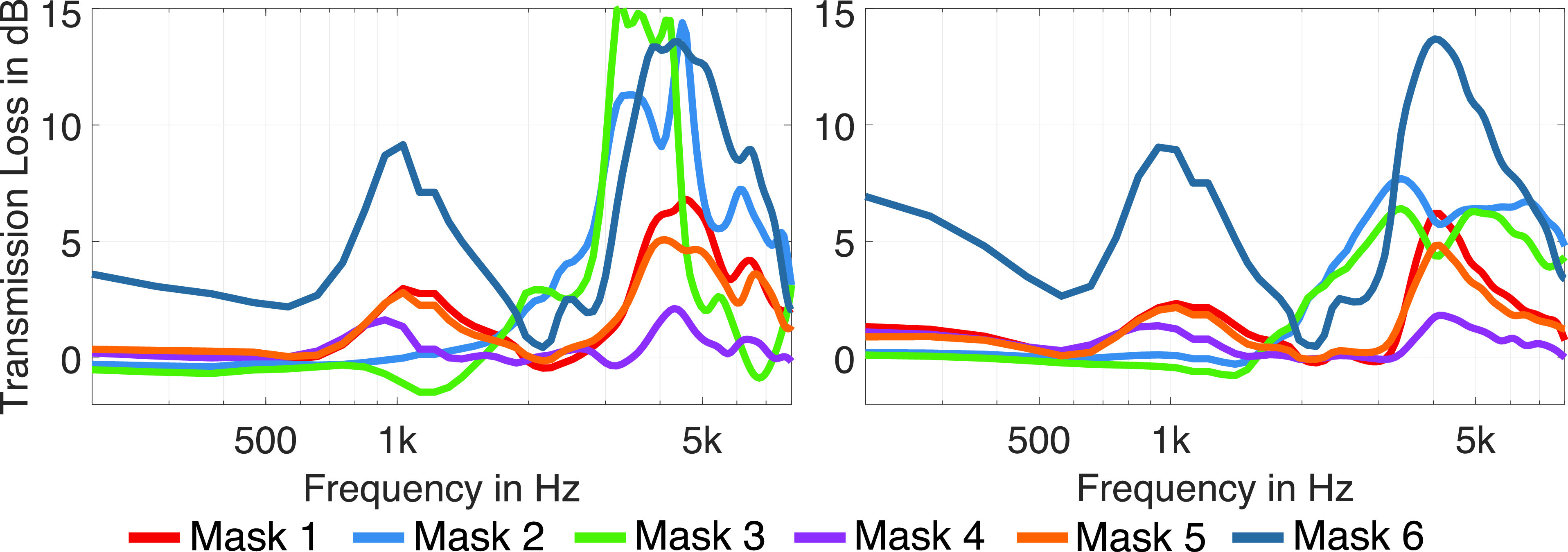
(Color online) Transmission loss $T(\Omega_{0},\omega )$, 1/12 octave smoothed, of the different masks for frontal sound radiation $\Omega_{0}=(\phi =0{}^{\circ},\theta =0{}^{\circ})$ (left), and averaged over all directions $T_{\text{avg}}(\omega )$ (right).

Figure 3 (left) shows the results for frontal sound radiation $\Omega_{0}$ = (ϕ=0°,θ=0°). Up to 2 kHz, the transmission loss is relatively low and flat for most masks but increases rapidly for masks 2 and 3 above 2 kHz with peaks of about 15 dB between 3 kHz and 5 kHz. Mask 6 shows a completely different frequency-dependent shape of the transmission loss. It exhibits a similar increase above 2 kHz, but already relevant transmission loss at low frequencies and a first strong peak at about 900 Hz.

For further analysis, we calculated $T_{\text{avg}}(\omega )$ by averaging the transmission loss over all radiation directions, *Q*,

$$T_{\text{avg}}(\omega )=10\text{lg}\frac{1}{Q}\sum_{q=1}^{Q}\frac{|p_{\text{ref}}(\Omega_{q},\omega ){|}^{2}}{|p_{\text{mask}}(\Omega_{q},\omega ){|}^{2}}.$$ (2)

Figure 3 (right) shows the results for the average transmission loss. In general, a similar trend can be observed as for the frontal sound radiation. However, for the medical respirator masks (masks 2 and 3), the peaks vary slightly with the radiation direction, and the shape of the curves becomes smoother and has less prominent peaks due to the averaging over direction. In contrast, for the other masks, the differences between Fig. 3, left and right, are much smaller, indicating that the peaks are rather independent of direction. Masks 1, 4, 5, and 6 show two peaks at about 900 Hz and 4 kHz, whereas for mask 4 the peaks are below 3 dB, and for masks 1 and 5, they are below 6 dB. Mask 6 exhibits peaks of more than 10 dB. Furthermore, for mask 6, the transmission loss is already above 3 dB at low frequencies. This may be due to the structure of this mask consisting of two layers of thick cloth.

### B. Directivity analysis

**FIG. 4.**
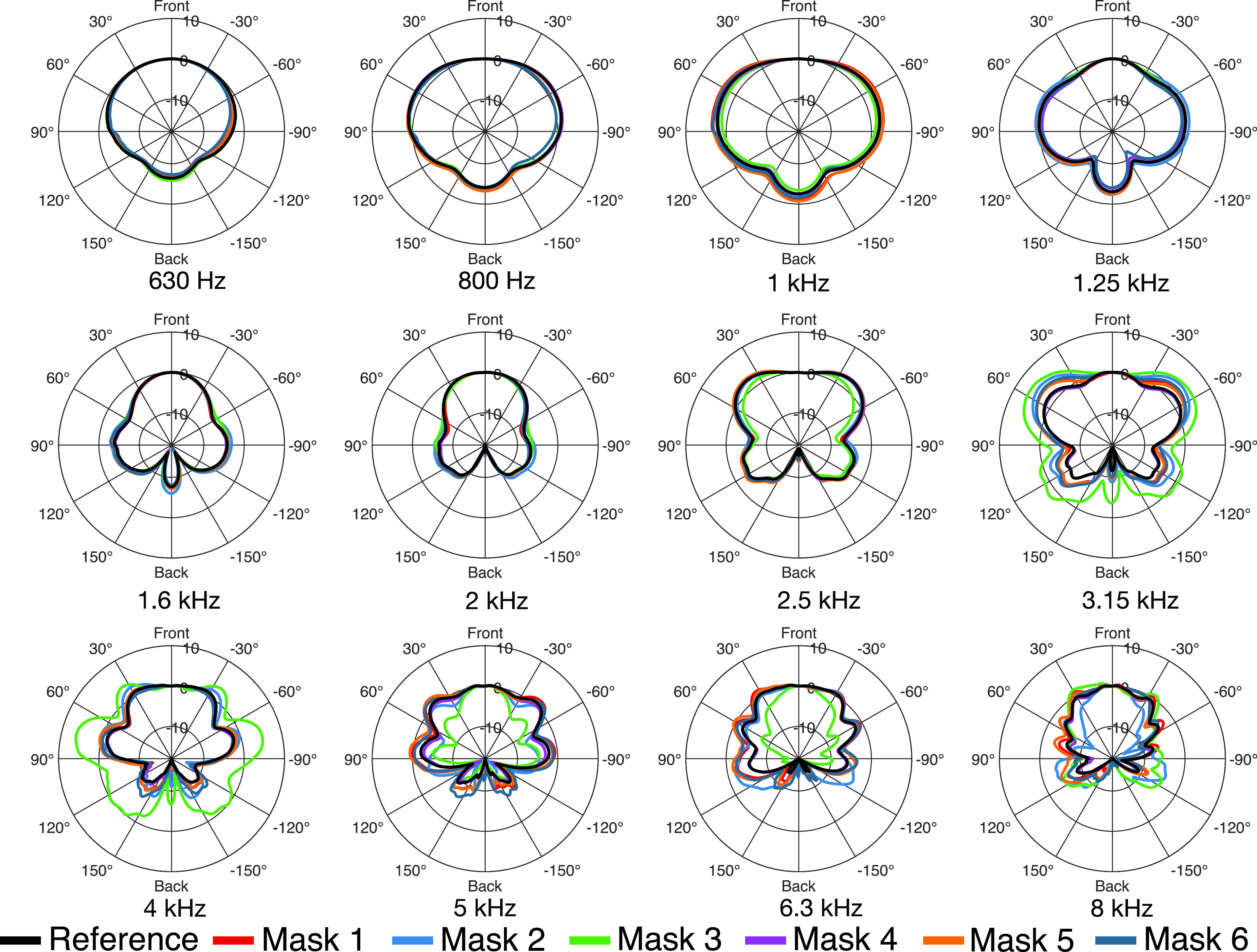
(Color online) Directivity patterns in the horizontal plane (ϕ=−180° to 180°,θ=0°) for all masks in third-octave bands.

**FIG. 5.**
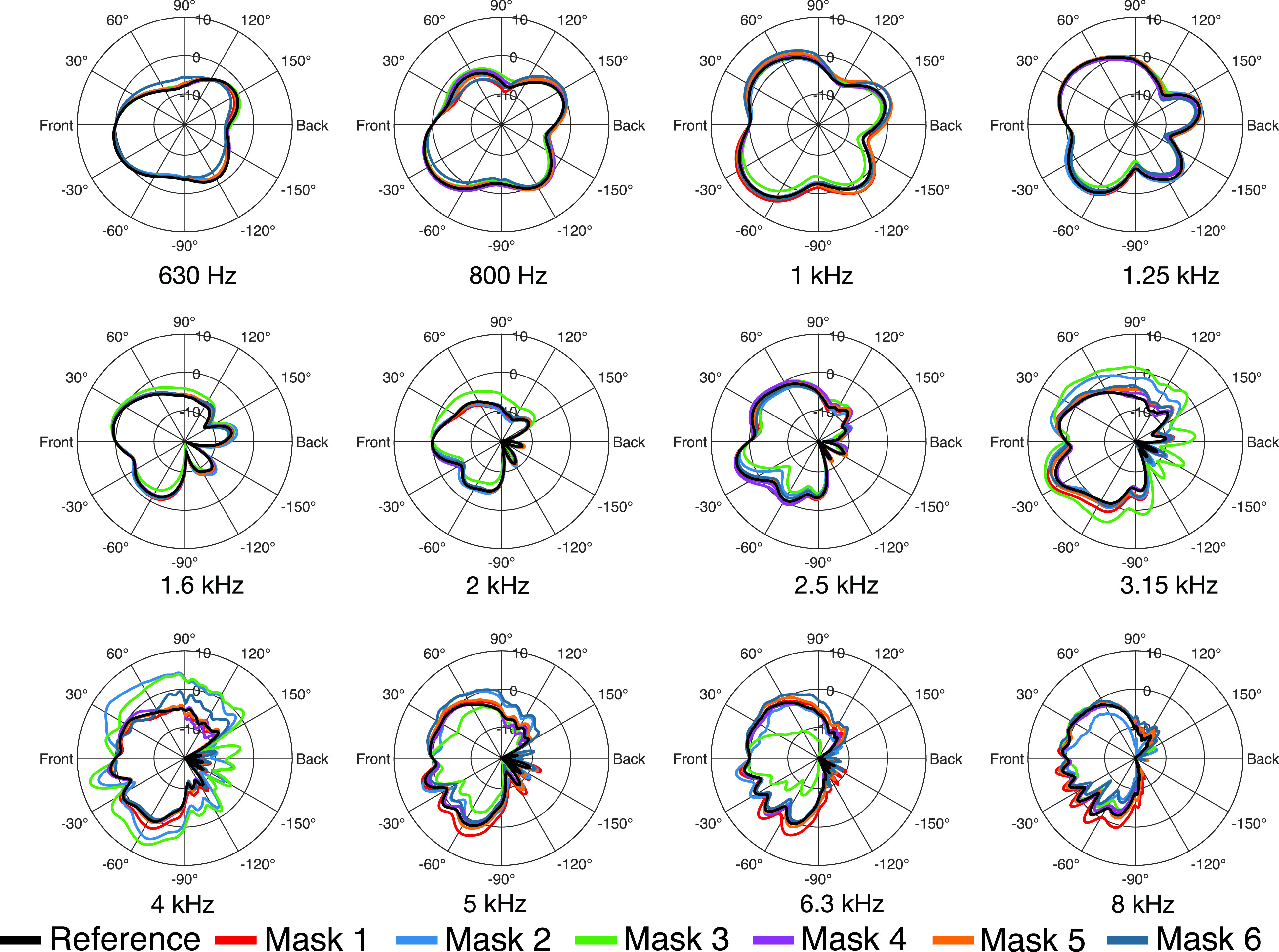
(Color online) Directivity patterns in the vertical plane (ϕ=0°,θ=−180° to 180°) for all masks in third-octave bands.

To analyze the directivity in the horizontal and vertical plane, the 2702 measured impulse responses were transformed to the spherical harmonics (SH) domain (Williams, 1999) at an SH order *N* = 35 and resampled to 360 directions in 1° steps along the horizontal plane [ϕ=−180° to 180° (where positive angles pointing to the left), θ=0°], and along the vertical plane [ϕ=0°,θ=−180° to 180° (where 0° points to the front, 90° to the top, and 180° to the back)] using the inverse SH transform. Figures 4 and 5 show polar plots of the horizontal and vertical directivities in third-octave bands. We only present the directivity patterns for the frequency bands above 500 Hz, as we could not observe any relevant influence of the masks on directivity for lower frequencies. For the third-octave bands up to 1.25 kHz, the directivities vary between the masks less than 1 dB, and thus, barley differ from the reference (black curve). Only for the directivity of mask 6 can some differences be observed, e.g., of 1 dB in the 630 Hz band at 60° in the vertical plane (Fig. 5). Above 1.25 kHz, the differences rise in magnitude with increasing frequency. As with the transmission loss, the filtering facepiece respirator masks (masks 2 and 3) exhibit the most prominent differences to the reference. In the 3.15 kHz band, which covers the same frequency range for which we observed the maximum transmission loss for the two masks, significant directivity variations occur. Furthermore, the directivities of masks 2 and 3 vary strongly for adjacent frequency bands. For example, the directivity at 4 kHz is much broader for mask 3 than for the other masks, while it is directed stronger to the front at 5 kHz. Please refer to the Appendix for a further illustration of the influence of the masks on sound radiation in the horizontal and vertical plane with respect to frequency.

### C. Directivity index (DI)

**FIG. 6.**
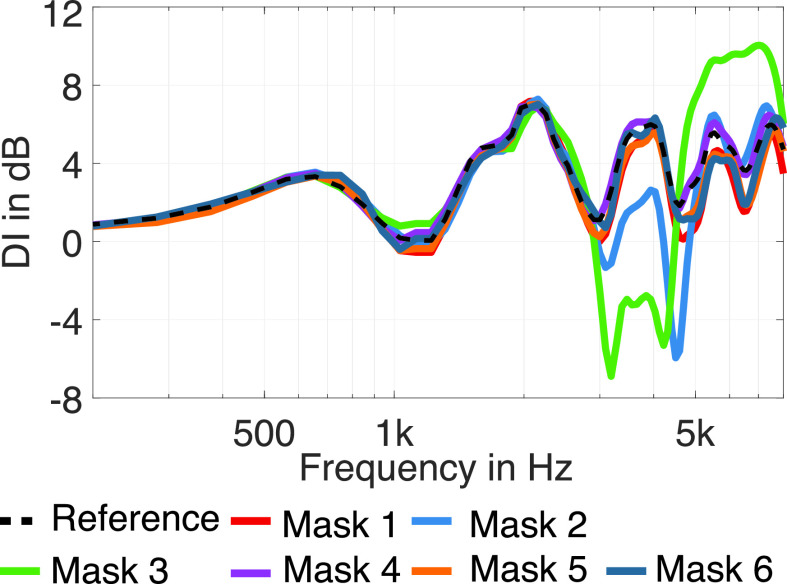
(Color online) DI, 1/12 octave smoothed, of the different masks.

For a more detailed analysis of the directivity, we determined the DI for spherical sound radiation, which can be calculated as

$$DI=10\;\text{lg}\frac{4\pi |p(\Omega_{m},\omega ){|}^{2}}{\int_{-\pi }^{\pi }\int_{-\pi /2}^{\pi /2}|p(\Omega ,\omega ){|}^{2}\;\text{cos}\;\theta d\theta d\phi },$$ (3)

where $\Omega_{m}$ denotes the main direction of sound radiation. Usually, the direction with maximum magnitude of radiation of the human mouth is inclined slightly downwards (Marshall and Meyer, 1985), which can also be seen in Fig. 5. However, we compensated the measurements for the frontal direction ($\Omega_{0}$), and furthermore, the frontal direction is included in all directivity plots. Therefore, we decided to calculate the DIs related to the main direction $\Omega_{m}=\Omega_{0}$. Table I summarizes the DIs for the third-octave bands between 630 Hz and 8 kHz. Apart from masks 2 and 3, the differences of the DI to the reference remain below 1 dB for frequencies up to 4 kHz and below 2 dB for frequencies up to 8 kHz. Again, the two facepiece respirator masks, masks 2 and 3, have the largest influence. In particular, between 3.15 and 6.3 kHz, the DI varies significantly. The differences are most prominent in the 4 kHz band, exceeding 4 dB (mask 2) and 7 dB (mask 3). This is supported by Fig. 6, which shows the DI with respect to frequency. In the frequency range from 3 to 5 kHz, the DI is significantly decreased by masks 2 and 3. At about 5 kHz the DI of mask 3 sharply rises towards high frequencies and is about 5 dB higher than the DI of all other measured datasets. The deviations of the DIs for the two facepiece respirator masks (masks 2 and 3) match the observations from Sec. III B, where we found the largest directivity variations for the same masks at the same frequencies (Figs. 4 and 5), and also the findings from Sec. III A, where we found the most considerable transmission loss for the corresponding masks in similar frequency regions.

**TABLE I.** DI in the third-octave bands related to the frontal direction $\Omega_{m}$.

Mask type	630 Hz	800 Hz	1 kHz	1.25 kHz	1.6 kHz	2 kHz	2.5 kHz	3.15 kHz	4 kHz	5 kHz	6.30 kHz	8 kHz
No mask	3.22	1.11	–0.42	1.86	4.99	6.60	3.72	3.54	4.97	4.00	4.47	5.69
Mask 1	3.43	1.18	–1.35	1.54	4.76	6.78	3.96	2.53	4.57	2.38	3.20	4.82
Mask 2	3.52	1.40	–0.44	1.18	4.55	6.50	4.21	0.42	0.93	4.12	4.93	7.21
Mask 3	3.23	1.26	0.48	2.09	4.75	6.14	4.88	–2.55	–2.29	7.86	9.65	6.47
Mask 4	3.38	0.93	–0.36	2.20	5.04	6.60	3.47	3.85	5.05	4.41	4.63	6.11
Mask 5	3.36	1.07	–1.22	1.58	4.69	6.57	3.65	2.79	4.43	2.80	3.33	5.22
Mask 6	3.55	1.46	–1.10	1.84	4.66	6.52	3.99	3.63	4.95	2.53	3.39	5.79

## IV. CONCLUSION

This paper presented measurements and analyses of sound radiation from a dummy head and mouth simulator wearing various face masks, i.e., medical masks, filtering facepiece respirator masks, and cloth face coverings. As discussed in Sec. II, a dummy head reproduces the typical spatial structure of human voice radiation, even though several aspects of human voice radiation like, e.g., the dynamic directivity, cannot be considered.

All examined masks result in a noticeable transmission loss at frequencies above 2 kHz. Thus, the transmission loss of the masks affects relevant frequency components of speech transmission, and therefore certainly impairs speech intelligibility. While in the frontal direction for the facepiece respirator masks (masks 2 and 3) the transmission loss increases strongly above 3 kHz by up to 15 dB, for the medical mask and most of the cloth face coverings, the transmission loss remains below 6 dB for frequencies above 3 kHz. An exception is a hand-made cloth face covering (mask 6), which consists of two thick layers of cotton, showing a much higher transmission loss already at low frequencies. Our results are in line with the results of Goldin *et al.* (2020). The study analyzed three different masks and determined an attenuation from 4 to 6 dB for a simple medical mask and an attenuation of 12 dB up to 18 dB for the higher protective N95 masks in the frequency range between 3 and 7 kHz.

The analysis of the directivity showed that the masks affect speech directivity differently. Both tested facepiece respirator masks lead to an increase of the DI by up to 7 dB between 3 and 5 kHz, and one of them to an increased DI for 5 dB above 5 kHz. The other masks show only a weak influence on the directivity, affecting the DI by a maximum of 1 dB for frequencies up to 4 kHz and 2 dB for frequencies up to 8 kHz. The DI can be directly related to the DRR, for which Larsen *et al.* (2008) determined just-noticeable differences (JNDs) of about 2–3 dB in rooms with a DRR of 0 or +10 dB and JNDs of about 6–8 dB in rooms with a DRR of −10 or +20 dB. For some frequency bands, the change of the DI caused by the facepiece respirator masks is in the range of the JND. Therefore, the facepiece respirator masks might impair speech intelligibility in rooms. However, as the DRR is a broadband measure, it is hard to predict how a decrease of the DI in specific frequency bands affects speech intelligibility. It can be assumed that especially phonemes incorporating significant energy in the affected frequency range might show degraded intelligibility. The datasets from this study can be applied in follow-up studies similar to Fogerty *et al.* (2020) or Kokabi *et al.* (2019) using room acoustic simulations or virtual acoustics to analyze in more detail which way the masks affect speech intelligibility.

## Figures and Tables

**FIG. 7.**
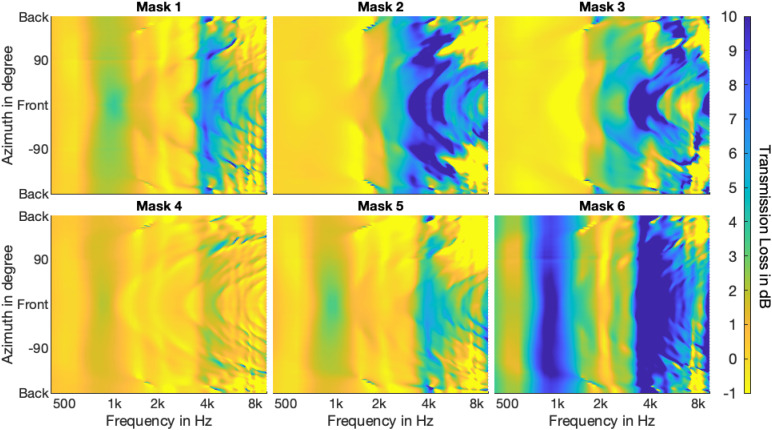
(Color online) Transmission loss T(ω) in the horizontal plane with respect to frequencies from 400 Hz to 10 kHz.

**FIG. 8.**
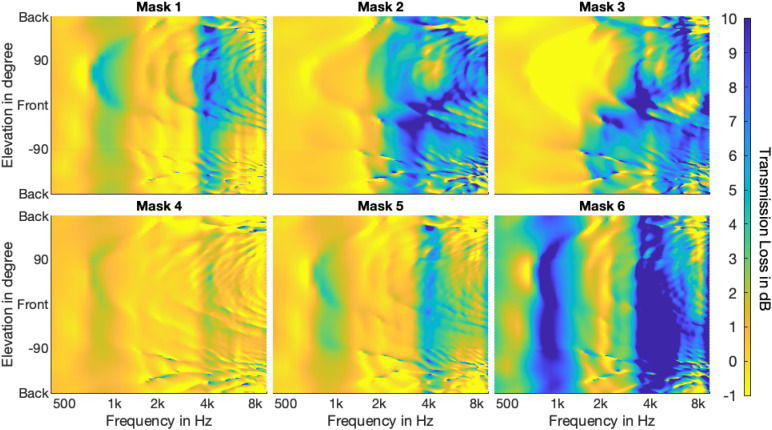
(Color online) Transmission loss T(ω) in the vertical plane with respect to frequencies from 400 Hz to 10 kHz.

## Footnotes

^1^ All directivity datasets are available in SOFA format under a Creative Commons CC BY-SA 4.0 license and can be downloaded at https://doi.org/10.5281/zenodo.3952320. The datasets will be published with a DOI on https://zenodo.org after acceptance of the paper.

## APPENDIX

Figures 7 and 8 show the transmission loss with respect to frequency in the horizontal and vertical plane for a frequency range of 400 Hz to 10 kHz. The figures illustrate the directional dependency of the transmission loss and support the observations from Secs. III A and III C. In particular, masks 2, 3, and 6 exhibit notable transmission loss, as indicated by the deep blue color.
